# Executive dysfunction and autobiographical memory retrieval in recovered depressed women^☆^

**DOI:** 10.1016/j.jbtep.2013.12.001

**Published:** 2014-06

**Authors:** Anneke D.M. Haddad, Catherine J. Harmer, J. Mark G. Williams

**Affiliations:** Department of Psychiatry, University of Oxford, Warneford Hospital, Oxford OX3 7JX, United Kingdom

**Keywords:** Depression, Recovered depressed, Autobiographical memory, Executive dysfunction, Female

## Abstract

**Background and objectives:**

Depressed individuals have difficulty remembering specific autobiographical events. These deficits often persist after recovery of mood symptoms, but the mechanisms underlying impaired memory specificity in recovered depressed individuals remain unclear. Here, we sought to examine whether performance on two cognitive measures might be related to deficits in autobiographical memory retrieval in individuals with a history of depression.

**Methods:**

Twenty-four recovered depressed women (12 with more than one previous episode) and 24 never depressed women completed two cognitive measures (Digit Span and a Number Generation Task) and tests of autobiographical memory recall.

**Results:**

Overall, the recovered depressed women did not show deficits in autobiographical retrieval. However, those with more than one previous episode had impaired retrieval of categorical autobiographical memories. Moreover, depression history moderated the relationship between Digit Span and retrieval of categoric autobiographical memories such that within the whole recovered depressed group (but not the never depressed group), those with lower Digit Span also had poorer retrieval of categorical autobiographical memories.

**Limitations:**

Our sample size was small and included only women. Moreover, order effects may have been a significant factor.

**Conclusions:**

These findings support the notion that working memory is an important factor in impairing autobiographical memory in those who have recovered from depression, but suggest a complex relationship with autobiographical recall.

## Highlights

• Recovered depressed women had no autobiographical memory (ABM) specificity deficits.
• Women with more than 1 past episode had categorical ABM deficits.
• In recovered depressed women, low categorical ABM correlated with worse Digit Span.
• The findings suggest a complex relationship between working memory and ABM.

## 1 Introduction

Depressed individuals have difficulties remembering specific autobiographical events. Whereas non-depressed people can describe single events that occurred at a particular time and place (e.g., ‘I was happy at my party last month’), depressed patients tend to describe repeated events (e.g., ‘I'm happy every year on my birthday’ – for a review, see Williams et al., 2007). In depression, this overgenerality is associated with deficits in important cognitive abilities – including reduced future specificity and poor social problem solving – and with delayed recovery from mood disturbances (e.g., Brittlebank, Scott, Williams, & Ferrier, 1993; Hermans, Vandromme, et al., 2008; Sumner, Griffith, & Mineka, 2010). Autobiographical memory specificity (AMS) deficits may continue after depressive symptoms have resolved (e.g., Mackinger, Pachinger, Leibetseder, & Fartacek, 2000; Nandrino, Pezard, Poste, Reveillere, & Beaune, 2002; Spinhoven et al., 2006), although there have been several failures to replicate this (Barnhofer, Crane, Spinhoven, & Williams, 2007; Wessel, Meeren, Peeters, Arntz, & Merckelbach, 2001; Williams, Barnhofer, Crane, & Beck, 2005). Moreover, in euthymic individuals, poor AMS may indicate vulnerability to future depression (Gibbs & Rude, 2004; Mackinger, Loschin, & Leibetseder, 2000; but see also Spinhoven et al., 2006). However, the cognitive mechanisms underlying reduced AMS remain unclear. Understanding these mechanisms could help improve interventions targeting depression and vulnerability to depression.

Several cognitive mechanisms have been suggested for AMS deficits, including functional avoidance and rumination (e.g., Williams, 2006). Here we focus on a third proposed mechanism: executive dysfunction (Dalgleish et al., 2007; Yanes, Roberts, & Carlos, 2008). Depression is associated with a range of executive deficits; such deficits may persist after recovery (Biringer et al., 2005; Kessing, 1998; Paelecke-Habermann, Pohl, & Leplow, 2005; Preiss et al., 2009). AMS, as assessed using the Autobiographical Memory Test (AMT), requires recall of specific autobiographical memories in response to cues. Satisfying the task requirements places considerable demands on various “executive” skills: to set verification criteria and hold them in working memory, to compare candidate memories with criteria, and to inhibit inappropriate responses. Poor AMT scores in depressed or recovered depressed individuals may thus be a direct consequence of deficits in executive capacity, broadly conceived.

In support of this account, several studies have found correlations between executive dysfunction and AMT scores (Dalgleish et al., 2007; Raes et al., 2006). Moreover, increasing the executive demands of the AMT or adding a cognitive load reduces AMS (Dalgleish et al., 2007; Williams et al., 2006). Finally, one study reversed the instructions of the standard AMT: Instead of requiring specific events, the task now required *categories* of events (Dalgleish et al., 2007, Study 8). Categoric responses represent errors on the standard AMT but correct responses on the reversed AMT. If depressive symptoms impair AMS *per se*, more depressed individuals would be more general, regardless of task instructions. However, if depressive symptoms impair the ability to fulfil task requirements, more depressed individuals would make more errors and thus would have poor *generality* on the reversed test. Consistent with the latter prediction, those with higher Beck Depression Inventory (BDI-II) scores gave more *specific* responses on the reversed test – consistent with the executive dysfunction explanation for autobiographical retrieval problems. Notably, in a separate study (Dalgleish, Rolfe, Golden, Dunn, & Barnard, 2008), use of the reversed AMT in a trauma-exposed sample resulted in the opposite pattern – that is, increased distress was correlated with reduced specificity even on the reversed test, suggesting a different mechanism underlying AMS deficits in depressed and trauma-exposed populations.

Dalgleish et al.s' (2007) participants were selected to have a range of BDI-II scores rather than on the basis of current or previous depression diagnosis. It remains unclear whether executive dysfunction can explain AMS deficits in recovered depressed individuals. As noted earlier, several studies have found persistent executive deficits in recovered depressed participants, but these have not assessed memory specificity. Moreover, most studies of persistent AMS deficits have not examined executive difficulties, though one study found that, amongst previously depressed individuals, better AMS correlated with better immediate and delayed recall (Spinhoven et al., 2006). However, executive measures were unrelated to memory specificity. Critically, this study did not describe how recovered depressed participants' executive capacity compared to that of controls. Moreover, some studies have found impaired executive abilities in those with recurrent, but not single, previous episodes (Kessing, 1998; Paelecke-Habermann et al., 2005). This is noteworthy in light of evidence for possible differences in AMS between single episode and recurrent patients, assessed when euthymic (Nandrino et al., 2002; Spinhoven et al., 2006), and suggests that the relationship between AMS and executive dysfunction in euthymic individuals with recurrent previous depression is worth examining further.

Here we sought to replicate and extend previous work by investigating autobiographical memory deficits in recovered depressed individuals. We tested euthymic previously depressed participants and never depressed controls on two measures of cognitive capacity, Digit Span and Number Generation Task, as well as on the standard and reversed AMT (Dalgleish et al., 2007). We hypothesized that, compared to the controls, the recovered depressed group would (a) have worse scores on the Digit Span and Number Generation Task; (b) produce fewer correctly specific memories on the AMT; and (c) produce fewer correctly categoric memories on the AMT-Reversed. We also predicted that the Digit Span and Number Generation Task would be correlated with the measures of autobiographical memory recall (both specific and categoric). A secondary aim was to examine whether those with recurrent previous depression show more exaggerated deficits on these measures.

## 2 Materials and method

### 2.1 Participants

Twenty-four recovered depressed but currently euthymic participants and 24 healthy controls participants were recruited from the local community. Only women were included because the recovered depressed participants took part in a further study (which has been reported separately, Haddad, Williams, McTavish, & Harmer, 2009) which sought to capitalize on women's increased sensitivity to effects of acute tryptophan depletion. All participants spoke English fluently.

On the basis of the Structured Clinical Interview for DSM-IV (SCID), recovered depressed participants met diagnostic criteria for at least one previous Major Depressive Episode, had not met criteria in the past six months, and did not meet criteria for any other past or current Axis I disorder. Recovered depressed participants had taken no antidepressant medication for at least three months and were not currently receiving psychotherapy. A recurrently depressed subgroup of 12 participants had more than one previous episode of depression. Controls had no current or past Axis I disorders and were not taking psychoactive medications or receiving psychotherapy.

All participants gave written informed consent and received £20 for their participation. The study was approved by the local Research Ethics Committee.

### 2.2 Measures

#### 2.2.1 Beck Depression Inventory

The BDI-II (Beck, Steer, & Brown, 1996) was used to assess presence and severity of depressive symptoms over the previous two weeks.

#### 2.2.2 Number Generation Task

Two versions of the Number Generation Task (Dalgleish et al., 2007) were used (A and B, each used with approximately half the participants); pilot testing showed no significant differences between versions. Participants were required to generate numbers that satisfied a set of constraints which were presented once verbally (e.g., “Please give me a sequence of five numbers between 499 and 101” or “Please give me a sequence of four numbers between 202 and 598”). Three practice questions were given (with feedback). Participants were informed that questions would be read only once. Errors were summed across types (numbers not in sequence, numbers outside the upper or lower bounds, incorrect number of numbers (e.g., giving five numbers, 101 through to 105, when asked for four numbers), incorrect number of digits (e.g., giving a seven digit number when asked for a six digit number)). Multiple instances of the same error type within the same sequence (e.g., giving six numbers when asked for four numbers, or giving two numbers below the lower bound) were counted as a single infringement of one constraint (Barnard, Scott, & May, 2001).

#### 2.2.3 Digit Span

Forward Digit Span was assessed by reading a list of digits at a rate of approximately one per second and asking the participant to recall them immediately in order. Initial lists were four digits long; if participants correctly recalled at least five out of six lists, the list length was increased by one. Digit span was the maximum length at which the participant correctly recalled at least five of six lists.

#### 2.2.4 Autobiographical memory tests

Two versions of the AMT were used: a standard version and a reversed version (AMT-Reversed). Forty cue words were divided into four lists of 10 words (five positive and five negative); lists were matched in terms of frequency, imageability, meaningfulness, concreteness, and familiarity using data from the MRC Psycholinguistic Database (University of Western Australia). In this study, two of the lists were used for each participant (one each for the AMT and AMT-Reversed). Previous testing with the AMT and AMT-Reversed in counterbalanced order in a separate sample of 50 participants (Haddad, 2008) revealed no significant order effects, so the AMT was always given first.

Participants were instructed to describe, for each AMT cue, a different memory of an autobiographical event lasting less than one day which had occurred at a specific time and place, and for each AMT-Reversed cue, a different memory of a category of events. Correct and incorrect examples were given. For each version, three neutral practice words were given (with feedback). Responses were recorded for subsequent coding. The outcome measures were the number of specific memories (for the AMT) or categoric memories (for the AMT-Reversed) that were given within 20 s^1^ of the cue and which did not repeat a previous response. Specific memories were defined as single episodes lasting less than a day and categoric memories were defined as events that had occurred on more than one occasion.

### 2.3 Statistical analysis

Analyses were performed in SPSS 20. Greenhouse-Geisser corrected values were used where the assumption of sphericity was violated. We used ANOVAs to compare the recovered depressed and control groups on the two AMT measures, with cue valence as a within-subjects factor. Between-groups *t*-tests were used to check for effects of depression history on the Digit Span and Number Generation Task. Finally, we used hierarchical linear regression to examine whether depression history moderated the relationship between each of these measures and autobiographical retrieval. The effect of recurrent depression was examined repeating each analysis comparing controls with the recurrent subgroup.

## 3 Results

### 3.1 Participants

Table 1 shows the characteristics of the participants. There were no significant differences between recovered depressed and control groups in terms of age or BDI-II scores, *p*s ≥ .233. There were also no significant differences in age or BDI-II score between the recurrently depressed participants and controls, *p*s ≥ .178. However, there were significant or trend-level differences in the *variance* of age and BDI-II between the recurrently depressed and control participants, so age and BDI-II score were included as co-variates throughout. Excluding these co-variates did not change the general pattern of findings. Mean age at depression onset in the recovered depressed group was 18.6 (SD = 4.3), mean number of episodes was 1.9 (range: 1–5), and mean time in remission at the time of participation was 2.8 years (SD = 2.2).

### 3.2 Autobiographical memory specificity

AMT scores for the recovered depressed and control groups were identical for both positive and negative cues; the mixed design (Valence × Depression History) ANOVA (with age and BDI-II score as covariates) yielded no significant effects, *p* values ≥ .460.

Using a similar analysis to compare AMT scores for the recurrent subgroup and controls also yielded no significant effects, *p* values ≥ .342.

### 3.3 Autobiographical memory generality

For AMT-Reversed scores, the mixed design (Valence × Depression History) ANOVA (with age and BDI-II score as covariates) yielded no significant effects, *p* values ≥ .075.

Repeating this analysis for the recurrent subgroup and controls yielded a main effect of depression history, *F* (1,44) = 4.417, *p* = .044, partial *η*^2^ = .121; other effects were not significant, *p* values ≥ .145. The effect of depression history arose because the recurrent subgroup gave fewer correctly categoric responses (M = 5.83, SD = 2.29) than controls (M = 7.08, SD = 1.76).^2^

### 3.4 Digit Span and Number Generation Task

Scores on the Digit Span and Number Generation Task measures were negatively correlated, *r* (48) = −.374, *p* = .009. There were no significant differences between the recovered depressed participants and the controls on either of these measures, *p* values ≥ .245. This remained the case when the recovered depressed group was restricted to those with recurrent depression, *p* values ≥ .239.

### 3.5 Autobiographical memory and Digit Span/Number Generation Task

To examine whether scores on Digit Span or Number Generation Task were differentially related to AMT scores in the recovered and never depressed groups, we tested for moderation effects using hierarchical linear regression. At stage 1, age and BDI-II score were added as covariates. At stage 2, depression history, Digit Span, and Number Generation Task errors were added, and at stage 3, the two interaction terms (Digit Span-by-depression history and Number Generation Task errors-by-depression history) were added. All predictors were mean centred before inclusion in the model. This analysis revealed no significant main effects or interactions.

**Fig. 1:**
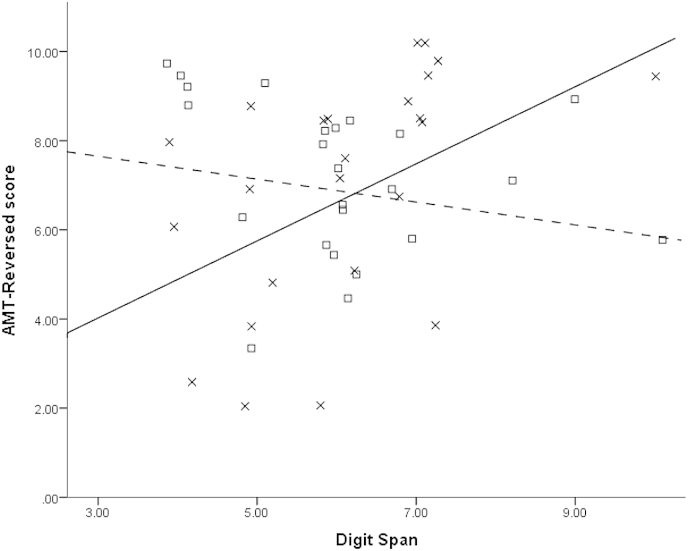
AMT-Reversed and Digit Span scores in the control (□) and recovered depressed (×) groups. Overlapping points have been offset slightly for clarity. The correlation is significant in the recovered depressed group (solid line) but not in the control group (dashed line).

We then performed a similar analysis for AMT-Reversed scores. This revealed no significant main effects of the predictor variables; however, inclusion of the interaction terms resulted in a significant stepwise *F* change, *F* (2, 40) = 3.557, *p* = .038, Δ*R*^2^ = .146. This resulted from the fact that depression history significantly moderated the relationship between Digit Span and AMT-Reversed score, *B* = .600, *t* = 2.360, *p* = .023; the moderation effect was not significant for Number Generation Task errors. Follow-up analyses to probe the origin of the moderation effect revealed that, whereas the partial correlation (controlling for age and BDI-II) between Digit Span and AMT-Reversed score was not significant for the control group, partial *r* (20) = −.224, *p* = .317, this relationship was significant for the recovered depressed group, partial *r* (20) = .497, *p* = .019. This indicated that amongst recovered depressed participants, those with better Digit Span also tended to have better scores on the AMT-Reversed (see Fig. 1).

## 4 Discussion

In this study we sought to replicate and extend previous work on autobiographical memory deficits in recovered depressed individuals. Three main sets of findings emerged. First, we found no evidence of impaired scores on the standard AMT in recovered depressed recovered depressed compared to never depressed individuals. Moreover, we found no evidence for impaired scores on the Digit Span and Number Generation Task. Second, however, those with a history of *recurrent* depression had impaired ability to retrieve categorical autobiographical memories on the AMT-Reversed. Third, impaired autobiographical memory generality was linked with poor Digit Span in recovered depressed individuals.

### 4.1 History of depression and memory specificity

We found no evidence of reduced AMS in recovered depressed participants; indeed, mean AMT scores for the two groups were exactly equal for both positive and negative cues. This contrasts with earlier studies demonstrating AMS deficits in recovered depressed participants (e.g., Mackinger, Pachinger, et al., 2000; Spinhoven et al., 2006) although several others have also failed to find this (Barnhofer et al., 2007; Wessel et al., 2001; Williams et al., 2005).

There are several possible reasons for our failure to replicate previous findings. First, our sample characteristics may have differed; half of our recovered depressed participants had only one previous episode whereas in Spinhoven et al.s' study (2006), recovered depressed participants had at least two episodes. Yet even when we used only the recurrent subgroup, there was no evidence of reduced AMS. Our power to detect AMS deficits in the recurrent subgroup may have been compromised by the small subgroup size. However, using G*power 3 (Faul, Erdfelder, Lang, & Buchner, 2007), we calculated that our power was ∼95% to detect an effect size comparable to Mackinger, Pachinger, et al. (2000) (Cohen's *d* = 1.05) or ∼74% for an effect size comparable to Spinhoven et al. (2006) (Cohen's *d* = .77).

A second possibility is that previous studies may have included individuals who, although not currently in a depressive episode, had residual depressive symptoms. In contrast, there were no significant differences in BDI-II between our groups. Additionally, we used a stringent definition of recovery: participants were non-depressed for at least 6 months and were not taking medications, whereas other studies included participants who had been well for shorter periods and who were taking antidepressants (Mackinger, Pachinger, et al., 2000; Spinhoven et al., 2006). These discrepancies highlight the need for further work characterizing factors that contribute to any ongoing AMS difficulties in recovered depressed individuals. Assuming that our failure to replicate previous findings does indeed arise because of differences in the sample characteristics, why would the recovered depressed individuals in our study have no longer exhibit impaired AMS? One possible explanation is that the underlying cognitive processes that impede retrieval specific memories are “dormant” during remission. For example, previous studies have shown that experimental inductions of particular thinking styles (self-discrepancy focus or abstract/analytical processing) in recovered depressed individuals can cause significant deterioration in the ability to retrieve specific memories, particularly in those with high trait rumination (Crane et al., 2007; Raes et al., 2012). In our study, however, these processes were not specifically activated and thus presumably were not interfering with retrieval of specific memories.

### 4.2 History of depression and memory generality

One aim was to examine whether recovered depressed participants are less able than controls to retrieve categoric memories on the AMT-Reversed. Previous work has shown that participants with more depressive symptoms give fewer categoric responses on the AMT-Reversed (Dalgleish et al., 2007). We therefore predicted that recovered depressed individuals would have ‘generality’ deficits on the AMT-Reversed. Although we found no evidence for impaired autobiographical memory generality on the AMT-Reversed in the recovered depressed sample overall, we did find a significant deficit in the recurrent subgroup, with a reasonably large effect size. To our knowledge, ours is the first study to demonstrate this deficit. This finding adds weight to the argument that autobiographical memory deficits in individuals with *recurrent* depression may be partly due to difficulties with following complex task instructions.

There are several possible reasons why this deficit may be limited to those with recurrent previous depression. One episode may not substantially impair the executive capabilities needed to perform the AMT-Reversed accurately. Alternatively, those with greater pre-morbid executive impairment may be more likely to have recurrent episodes, or some third factor may cause both. For example, earlier onset may signal both increased risk of recurrence and greater risk of ‘scarring’. Further research is needed to examine these possibilities, ideally using prospective designs.

Why were deficits in recurrently depressed individuals found on the AMT-Reversed but not the standard AMT? One possibility is that the AMT-Reversed requires more unusual responses and is therefore less ecologically valid. We would argue, however, that retrieving categorical autobiographical memories is an important skill in daily life. For example, one might recall “Whenever I go to work via Piccadilly Circus, I get there late” or “When I forget to send my mother a birthday card, she gets upset.” A person with deficits in retrieving such categoric memories might be less adept at solving the problems evident in these situations. Our data suggest that individuals with a history of recurrent depression are not as good at retrieving such categoric memories *on demand* in the laboratory and, perhaps, would also be impaired in retrieving categoric memories *when the situation demands it* in daily life. As noted by Williams (2006, p. 550), “Fluent access to [both specific and categoric memory types] is required for skilful navigation through the complexities of the world, especially the interpersonal world”. Being able to retrieve either specific or categoric autobiographical memories in a *flexible* fashion is probably optimal (Hermans, de Decker, et al., 2008). However, further work is needed to examine whether lower scores on the AMT-Reversed are associated with poorer social problem solving or with other important impairments of social and emotional wellbeing.

Another possible interpretation of the differential findings between the AMT and the AMT-Reversed is that this reflects perseveration errors or order effects arising from the fact that the AMT was always administered first. That is, impaired performance in recurrently depressed participants could reflect difficulty with switching mental set and consequent continuation of responding according to the AMT instructions. If this were the case, errors on the AMT-Reversed would take the form of *overspecific* errors rather than any other sort of error. Yet there were no differences in number of incorrectly specific responses on the AMT-Reversed between the recurrent and control groups, *t* (11.613) = 1.289, *p* = .222 (corrected for unequal variance), indicating that the AMT-Reversed impairment in recurrently depressed participants was unlikely to be due to perseveration. However, order effects may nevertheless have contributed to the findings. For example, 'self-regulation' depletion has been shown to reduce memory specificity on the standard AMT (Neshat-Doost, Dalgleish, & Golden, 2008). Insofar as this might also apply to the AMT-Reversed, and if self-regulation is depleted more rapidly in recurrently depressed individuals than in never depressed individuals, it is plausible that, as the study went on, recurrently depressed individuals had more severe self-regulation depletion than controls – with consequent deleterious effects on the AMT-Reversed which was administered later in the session. However, this suggestion is necessarily speculative; our findings regarding the AMT-Reversed must remain tentative until they have been replicated and possible mechanisms explored empirically.

### 4.3 History of depression, autobiographical memory retrieval, and Digit Span

Recovered depressed participants showed no deficits on the Digit Span or Number Generation Task and there was no relationship between AMT scores and scores on these measures However, Digit Span and AMT-Reversed score were significantly correlated amongst recovered depressed participants, but not controls. This suggests that Digit Span may be protective against deficits in retrieving categoric autobiographical memories. Given our earlier finding that those with repeated episodes of depression have worse performance on the AMT-Reversed, it may therefore be of interest to examine whether improving working memory might improve *flexibility* of autobiographical retrieval, enabling individuals to retrieve memories that are more appropriate to a given set of situational demands.

It is unclear why this finding was specific to the Digit Span measure and was not found for the Number Generation Task. Digit Span is a more generic measure of working memory capacity, in that material is highly-overlearned, so that only the *quantity* of material varies. Moreover, Digit Span instructions are straightforward and unchanging, so any confusion is *within* each to-be-remembered item (e.g., numbers in wrong order). In contrast, confusion in the Number Generation Task mainly arises from interference at the level of the instructions (i.e., between the current and previous items); this adds to working memory load, because participants need to retain a complex and changing array of instructions. It might be argued that the AMT and AMT-Reversed involve unvarying instructions, so retrieving instructions is unproblematic, but a reduction in generic working memory is sufficient to affect retrieval. Indeed, a previous study of impaired AMS pointed to the possibility that impaired memory specificity arise from difficulties with retaining the task instruction set in working memory even though no mental operations on the content are necessary (Yanes et al., 2008).

### 4.4 Limitations

Several limitations must be acknowledged. Firstly, our sample was small and only included women. Moreover, although our overall sample was adequately powered to detect effect sizes comparable to previous studies, the recurrent subgroup was particularly small, so our power to detect AMS deficits in this group may have been limited. Secondly, we did not measure levels of traumatic symptomatology. A history of trauma is associated with decreased AMS, although depression and trauma history are confounded in many studies (Williams et al., 2007). Moreover, as noted earlier, the mechanisms underlying overgeneral memory in depression and trauma may be different, with higher trauma-related distress being associated with *reduced* specificity on the AMT-Reversed despite the opposite pattern being observed in depressed/dysphoric individuals (Dalgleish et al., 2008). Our exclusion of those with current or past post-traumatic stress disorder means that any confounding of depression with this condition should not have substantially influenced our results. Yet this means our findings may not generalize to those with memory deficits due to trauma rather than depression. Moreover, our participants were relatively young women with relatively few previous episodes, which may again limit the generalizability of findings. Finally, as noted earlier, the possibility that order effects may have affected the AMT-Reversed findings cannot be excluded. However, even if these results were entirely due to order effects, then it is a non-trivial finding that such effects can compromise measures of autobiographical memory retrieval in recovered depressed but not never depressed groups.

### 4.5 Future work

These findings might inform future work in this area in a number of ways. Firstly, further clarity is needed regarding the factors that influence the presence or absence of memory specificity deficits in recovered depressed individuals, with particular consideration of number of previous episodes, presence of residual symptoms, and activation of particular thinking styles – and how these factors might interact. Secondly, further examination of the role that self-regulation depletion may play in impairing autobiographical recall in recovered and never depressed individuals is warranted. This could be done by administering tests of autobiographical recall to such participants immediately after a cognitively demanding task, such as the Stroop task, with the expectation that this might result in lower scores in recovered depressed but not never depressed individuals. It might be particularly interesting to investigate the possibility of differential outcomes depending on whether the recall task was the AMT or the AMT-Reversed. Finally, investigating the relationship between working memory and autobiographical recall may prove fruitful. For example, studies manipulating working memory load in recovered depressed individuals whilst they retrieve categoric autobiographical memories (e.g., adding a cognitive load, or training to improve working memory) would shed more light the mechanisms underlying autobiographical retrieval difficulties in recovered depressed groups. Related to this, it may be interesting to examine how Mindfulness Based Cognitive Therapy, which both improves AMS and reduces the risk of relapse in recovered depressed individuals (Teasdale et al., 2000; Williams, Teasdale, Segal, & Soulsby, 2000), affects working memory capacity.

### 4.6 Conclusion

In summary, we were unable to replicate previous findings showing impaired retrieval of specific autobiographical memories and persistent executive dysfunction in recovered depressed individuals. However, we found that *recurrent* previous depression is associated with impaired retrieval of categoric autobiographical memories. Moreover, we found that amongst recovered depressed participants, those with worse Digit Span had poorer retrieval of categorical memories. These results substantiate the view that working memory is an important factor in impairing autobiographical recall, but suggest that the relationship is complex. Further work is needed to examine how these factors are affected by treatments designed to reduce risk of future depressive episodes in recovered depressed patients.

## Statement of interests

CJH is on the advisory board of P1vital and has received consultancy fees from Servier, P1vital, Lundbeck, and Merck-Sharpe and Dohme. She holds shares in P1vital Ltd and is a company director of Oxford Psychologists Ltd.

## Figures and Tables

**Table 1:** Characteristics and scores on the memory and executive measures for the control and recovered depressed groups, and for the recurrently depressed subgroup.

	Control (*n* = 24)			Recovered depressed (*n* = 24)			Recurrently depressed subgroup (*n* = 12)		
	Range	Mean	SD	Range	Mean	SD	Range	Mean	SD
Age	19–39	24.58	4.84	18–54	26.46	8.22	19–54	29.08	10.43
BDI (II) score	0–13	3.04	3.14	0–21	4.38	4.39	0–21	4.75	5.85
AMT score (positive cues)		4.13	.95		4.13	1.03		4.33	.89
AMT score (negative cues)		3.79	1.28		3.79	1.38		3.50	1.51
AMT-Reversed score (positive cues)		3.63	1.44		3.46	1.41		2.83	1.47
AMT-Reversed score (negative cues)		3.46	.98		3.46	1.35		3.00	1.21
Number Generation Task errors		3.13	2.58		4.21	3.69		4.67	4.08
Digit Span		6.04	1.49		6.08	1.35		5.83	1.11

## Footnotes

1 Although it is more common to use a cut-off of 30 or 60 s, we used 20 s because this was the cut-off we used in a previously published paper which used the same recovered depressed participants as the current study (Haddad et al., 2009). However, audio recordings of the participants' responses allowed us to obtain scores using a 30 s cut-off; repeating the analyses using these scores did not change the overall pattern of findings.

2 Note that, if age and BDI-II are not controlled for, the simple between-groups *t*-test is only at trend level, *t* (34) = 1.812, *p* = .079, Cohen's *d* = .61.
